# Nutritional care for cancer patients: are we doing enough?

**DOI:** 10.3389/fnut.2024.1361800

**Published:** 2024-04-24

**Authors:** Valentina Da Prat, Paolo Pedrazzoli, Riccardo Caccialanza

**Affiliations:** ^1^Clinical Nutrition and Dietetics Unit, Fondazione IRCCS Policlinico San Matteo, Pavia, Italy; ^2^Department of Internal Medicine and Medical Therapy, University of Pavia, Pavia, Italy; ^3^Department of Oncology, Fondazione IRCCS Policlinico San Matteo, Pavia, Italy

**Keywords:** cancer, nutritional oncology, anticancer diets, nutritional support, malnutrition

## Abstract

Malnutrition is associated with higher rates of surgical complications, increased anticancer treatment toxicities, longer hospital stays, higher healthcare costs, poorer patient quality of life, and lower survival rates. Nutritional support has been shown to improve all of these outcomes. However, the nutritional care of cancer patients is still suboptimal and several issues remain unresolved. Although the effectiveness of nutritional support depends on the timeliness of intervention, assessment of nutritional status is often delayed and perceived as unimportant. When diagnoses of malnutrition are made, they are rarely recorded in medical records. Hospitals lack medical staff dedicated to clinical nutrition, making it difficult to integrate nutritional care into the multidisciplinary management of cancer patients. Outside the hospital, nutritional support is hampered by heterogeneous reimbursement policies and a lack of adequate community nutrition services. In addition, an increasing number of patients are turning to potentially harmful “anti-cancer” diets as trust in medicine declines. Adopting mandatory nutrition screening, monitoring quality of care metrics, providing nutrition education to care providers, and implementing telehealth systems are some of the most urgent interventions that need to be established in the future.

## 1 Introduction

Malnutrition is a harmful condition resulting from deficiencies, imbalances or excesses in a persons’ intake of nutrients and/or energy (1). In the common sense of the word, malnutrition is usually considered to be undernutrition, when the intake of energy and nutrients is insufficient to meet the individual’s needs. This condition is particularly common in patients with various chronic and acute diseases, including cancer (2). It has been estimated that up to 14% of patients with stage IV neoplasia are malnourished, while 49% of them are at risk of malnutrition according to validated screening tools (3). Many factors contribute to the unfavorable energy balance in these patients, including deregulation of systemic inflammatory pathways leading to anorexia, reduced energy intake, increased muscle catabolism, lipolysis and acute-phase protein synthesis, and impaired gastrointestinal function due to tumor involvement and/or treatment toxicities (4).

It is well known that malnutrition is associated with a higher incidence of surgical complications, increased anticancer treatment toxicities leading to reduced chemotherapy dose intensity, longer hospital stays, higher costs, poorer patient quality of life, and lower survival rates (5).

Nutritional support has been shown to improve all of these outcomes, including survival (6) and is now included in all major international cancer care guidelines (7).

However, the nutritional care of cancer patients is still suboptimal and several issues remain unresolved. In this Perspective, we focus on key challenges related to health care systems in high-income countries and propose possible solutions to improve malnutrition management in oncology.

## 2 Nutritional assessment in routine clinical practice

The basis of nutritional management in cancer is the early identification of patients at risk of malnutrition and, which should be performed using validated screening tools, according to the GLIM approach (8).

Screening tools are well known and require minimal training to be used, but there is no international consensus on the best tool to use in specific patient populations, including cancer patients. The Global Leadership Initiative on Malnutrition (GLIM) recommends the use of *any* validated screening tool, which include the Malnutrition Universal Screening Tool (MUST), the Malnutrition Screening Tool (MST), the Nutritional Risk Screening 2002 (NRS-2002), the Patient-Generated Subjective Global Assessment-Short Form (PG-SGA SF), and the Mini-Nutritional Assessment Short Form (MNA SF) (8). Similarly, guidelines from international nutrition societies do not provide any specific indications on the preferred tool to use, although they are consistent in recommending nutritional screening for cancer patients (9). For example, the European Society for Clinical Nutrition and Metabolism (ESPEN) suggests that inadequate nutritional intake, weight loss, and low body mass index (BMI) should be considered in nutritional risk screening (10), and the American Society for Enteral and Parenteral Nutrition (ASPEN) recommends malnutrition screening but does not specify which tool to use (11). The lack of consensus on the best tool to use results in high heterogeneity of malnutrition screening modalities across countries and sometimes within the same country, with difficulties in comparing the results of clinical trials on malnutrition.

Studies assessing the accuracy of nutritional screening methods for outpatients with cancer were recently reviewed (12). Interestingly, the performance of screening tools was found to be highly dependent on tumor type. For example, MUST was found to perform better in patients with digestive tumors, while NRS-2002 identified a higher prevalence of malnutrition risk in patients with hematopoietic tumors. This may depend on the different features of each screening tool; for example, some tools include disease burden (NRS-2002), functional capacity (MNA SF, PG-SGA SF), or neuropsychological problems (MNA SF), while others do not; some tools require trained health professionals to administer them (MUST, NRS-2002, MNA SF) while others do not (PG-SGA SF, MST) (13).

Besides the lack of a standardized procedure for nutritional screening and the pitfalls of each tool, nutritional screening in cancer patients is rarely incorporated into routine clinical practice in oncology departments. For instance, in 2020 a survey conducted by our group showed that nutritional assessment was performed at diagnosis by only 27% of Italian oncologists, while validated nutritional screening was used in only 16% of oncology units (14). Moreover, a European survey including more than 900 cancer patients and survivors showed that only 35% of respondents reported having their weight measured regularly during treatment, while 46% believed that their physician considered cancer-related weight loss unimportant (15). Reduced use of malnutrition screening has also been described in the United States (U.S.), where only 53% of outpatient cancer centers reported screening for malnutrition risk, and of these, only 65% used a validated screening tool (16). Nevertheless, economic impact analyses have shown that malnutrition screening is sustainable and potentially beneficial to hospitals by reducing malnutrition-related morbidity and length of hospital stay (17). In addition, nutritional screening at diagnosis allows for early implementation of nutritional support, in a stage of cancer-related malnutrition t where an anabolic window exists (18). Although only few studies have succeeded in demonstrating a survival benefit with nutrition support in the oncology setting, in the secondary analysis of the EFFORT trial early supplementation was shown to significantly reduce 30-day mortality and to improve quality of life and functional status of inpatients with cancer (6, 19).

## 3 Malnutrition diagnoses in medical records

Diagnosis-related group (DRG)-based hospital payment systems have gradually become the principal means of reimbursing hospitals in many countries across the world, including many European states, Australia, China, Japan, and the United States, although there are differences in cost accounting between countries (20, 21). In the DRG system, episodes of hospitalization are classified into specific case groups, represented by a DRG code, based on the primary and secondary diagnoses and procedures documented in the medical record. Hospital reimbursement is based on the average cost of caring for patients with diagnoses of that DRG code, regardless of the actual cost of the patient’s stay; the reimbursement value increases depending on the coding of secondary diagnoses that reflect major complications and comorbidities (MCCs) or complications and comorbidities (CCs) associated with the care of that patient. Malnutrition, depending on its severity and corresponding International Classification of Diseases, 10th revision (ICD-10) classification, can qualify as both an MCC (in case of cachexia, ICD-10: R64) or a CC (in case of protein-calorie malnutrition, ICD-10: E40-E46) (22). In particular, in the absence of other complications, the diagnosis of malnutrition can turn an uncomplicated DRG diagnosis into a DRG with CC or MCC and thus increase reimbursement.

Studies conducted in different countries demonstrate that the rate of malnutrition diagnosis in medical records is largely suboptimal and that simple educational interventions to improve malnutrition identification and coding, if performed, can lead to significant economic benefits. In a recent Chinese study, the inclusion of malnutrition among DRG diagnoses resulted in a change in complication group in 44% of malnourished patients compared to the pre-intervention period (23). Similarly, an educational intervention on nutritional assessment and coding in the Medicare Severity-DRG system in the U.S. led to a threefold increase in reimbursement for malnourished patients (22). In Belgium, a study evaluating the impact of optimizing malnutrition assessment showed a 9-fold increase in economic revenue after the intervention (24). The low coding rate of malnutrition and the associated loss of reimbursement was confirmed in a Spanish study on 266 oncology patients, where the diagnosis of malnutrition was not coded in one-third of the patients, resulting in more than 191,000€ not being reimbursed to the hospital (25). Moreover, the favorable economic impact of malnutrition diagnoses on hospital reimbursement appears to be sustainable since the costs of nutritional assessment and therapies are covered by the economic surplus generated by coding malnutrition, as shown in a Swiss study on more than 169,000 inpatients, nearly one-third of whom had a cancer diagnosis (26).

In addition to economic considerations, the inclusion of malnutrition in the list of diagnoses could help to emphasize the role of nutritional status as an important part of the patient’s clinical picture and encourage early nutritional assessment and timely initiation of nutritional support measures during future hospitalizations.

## 4 Staff dedicated to clinical nutrition

There is great heterogeneity among countries regarding the different professionals involved in providing nutrition care. For example, in the United States, registered dietitians (RDs) play a critical role in the prescription of artificial nutrition, but in many European countries, including Italy, only physicians can prescribe enteral and parenteral nutrition, while RDs provide dietary counselling and oral nutritional supplementation under medical supervision. In addition to dietitians and physicians, several countries have other professionals working in the nutrition field, such as nutritionists, specialized nurses and pharmacists. Concerning medical personnel, some countries offer specific residency programs in clinical nutrition, while in other countries (e.g., the U.S.) physicians with different backgrounds (e.g., surgery, intensive care, and gastroenterology) follow specific nutrition programs after residency. Often, clinical nutrition specialists work in other support areas (e.g., internal medicine wards or emergency departments), especially if they have previously worked as anesthesiologists, gastroenterologists or internal medicine physicians; a European survey showed that only 12% of physicians in nutrition support teams were solely responsible for nutrition (27). Overall, a low level of nutrition education in medical universities has been described (28), which may have resulted in few medical students choosing clinical nutrition as a postgraduate career in the past, and many physicians entering the field of nutrition after years in other medical professions.

Overall, the number of clinical nutrition personnel seems to be insufficient to meet the needs of the population due to the results of previous planning, socio-economic factors and, above all, the impact of the COVID-19 pandemics, which have adversely affected the quality of health care in economically developed countries (29). According to the literature, 30–66% of cancer patients report unmet nutritional information needs (30), and a recent Spanish survey showed that although 86.6% of healthcare providers reported that nutritional information was provided to patients, only 33.5% of patients reported having received it (31). Moreover, a U.S. survey of 215 cancer centers showed an RD-to-patient ratio of 1:2,308; in this study, RDs evaluated and counselled an average of 7.4 ± 4.3 oncology patients per day, reflecting a gap in RD access for oncology patients requiring nutrition care (16).

## 5 Implementation of multidisciplinary management

In the last decades, the multidisciplinary collaboration of different professionals has been shown to be essential and fruitful in cancer care: in national and international scenarios, multidisciplinary working groups have been established, including oncologists and clinical nutritionists, while at the hospital level, multidisciplinary tumor boards (MTBs) have been implemented (7, 32). Although MTBs are an essential element of multidisciplinary applied to cancer care, their constitution might be difficult, especially outside major academic institutions, in rural areas and in less well-structured healthcare systems. In the literature, many studies have demonstrated the benefits of MTBs in terms of improved care processes, increased adherence to guidelines, improved cancer outcomes, increased attention to patient perspectives, competence development, and cost control (32). MTBs include members of various specialties, with a core of surgeons, clinical and radiation oncologists, radiologists, and pathologists; however, clinical nutrition specialists are rarely included or even mentioned. Nevertheless, several aspects of cancer care, including decisions regarding the timing of interventions, the need for enteral/parenteral access implantation, and access to palliative care could benefit from the exchange of views and perspectives with nutrition specialists and fit perfectly with the goals of the MTBs. Unfortunately, due to the small number of nutrition specialists, it is often not possible to include them in multidisciplinary committees, with the risk of disruption to committee schedules or excessive time away from patient care.

## 6 Reimbursement policies for home nutrition care

Although nutritional care often begins as a hospital-based intervention, it evolves in the community. Many nutritional therapies have been shown to be effective only if provided over a long period: for example, oral nutritional support is associated with higher long-term survival rates in medical patients if supplementation is continued after hospital discharge (33). However, it is very difficult to ensure the distribution of oral nutrition products to all cancer patients at nutritional risk due to heterogeneous reimbursement policies and the lack of an adequate number of community nutrition services. In particular, both in countries with insurance-based systems (e.g., United States) and in countries with public healthcare systems (e.g., Italy), patients are at risk of being denied home nutritional support, e.g., in the case of lower-paying insurance plans, low regional government reimbursement, or reduced availability of home care providers, resulting in disparities based on patient economic status or regional origin.

A recent study by the Professional Society for Health Economics and Outcomes Research (ISPOR) characterized the coverage and reimbursement of medical nutrition, defined as food for special medical purposes/medical food, for several countries, including Australia, Belgium, Brazil, Canada, China, France, Germany, Hong Kong, Italy, Japan, the Netherlands, Singapore, Spain, the United Kingdom, and the U.S. (34). Medical nutrition was usually covered in the hospital setting (14/15 countries), while only 9 of the 13 countries that provided coverage in the outpatient setting and 8 of the 12 countries that provided coverage in the community setting limited coverage to specific subsets of patients (e.g., patients with low income, malnourished patients, or specific populations such as retired army veterans). Only France, Germany, Spain, and the Netherlands provided coverage both in all of inpatient, outpatient and community settings. In addition, this study highlighted that despite innovations in nutritional formulations with improved outcomes and stronger evidence of their effectiveness, the underlying logic of reimbursement policies in most countries has remained largely unchanged in the last 20 years, with the evolution of reimbursement policies disconnected from the evolution of clinical guidelines. As a result, for some individuals excluded from reimbursement, suboptimal nutritional care could lead to increased morbidity, decreased quality of life, increased healthcare costs, and increased mortality (34).

## 7 Patient trust in medicine

Overall, there is a perception among many researchers that public trust in nutrition science is eroding. Loss of public trust is likely due to several factors, including the increasing complexity of modern nutrition science, the perception that experts are constantly changing their assessments of the available evidence, and the polarization of public debate on scientific issues (35, 36).

Conversely, the proliferation of potentially harmful dietary approaches is facilitated by the spread of misconceptions and self-appointed “experts,” who find fertile ground in the absence of easily accessible clinical nutrition services and in the ready availability of means of communication (web, social media) where quality control is lacking. In addition, the promotion of non-science-based treatments and fraudulent claims often takes advantage of a public that is unable to evaluate the quality and accuracy of the information provided (37, 38).

In oncology, this often translates into patients adhering to harmful diets with putative anti-cancer effects and/or self-administration of nutritional supplements with little scientific evidence of efficacy. In particular, “anti-cancer” dietary regimens often consist of restrictive diets (e.g., fasting, ketogenic diets, unbalanced vegan diets) which can compromise a patient’s nutritional status if not monitored (39). In the NutriNet-Santé study on more than 2,700 cancer survivors, 6% of patients had fasted in an attempt to improve their cancer prognosis; compared to patients who did not fast, fasting patients were less likely to have received nutritional information from health professionals (40).

The contribution of emotional factors should not be neglected when analyzing patients’ adherence to non-science-based diets. From a psychological point of view, self-prescribed dietary restrictions may be a consequence of the feelings of powerlessness and overwhelm that many cancer patients experience due to their uncertain clinical course. This often leads to the need to regain a sense of mastery and to create predictability in their daily routines through dietary management, over which patients feel a sense of control (41). Unmet psychological needs and lack of psychological and communication training among nutrition care providers are likely to contribute to patients’ vulnerability to potentially harmful reference points on the Internet and social media.

## 8 Discussion

Nutrition care is essentially a long-term investment. As such, it requires strategic foresight to anticipate its future benefits—a challenging task in an era of quick thinking and resource optimization. In this Perspective, we have focused on several issues still to be solved in the field of nutritional care for cancer patients, with the goal of raising awareness of the barriers to implementing optimal care in clinical practice and laying the groundwork for possible solutions.

To optimize the use of nutritional screening, every effort should be made to implement awareness campaigns focusing on the importance of early nutritional care. Patient advocacy could play a key role in reminding institutions that nutritional care is a right and not an option for cancer patients (42).

The introduction of mandatory nutritional screening for cancer patients should be considered, possibly involving other members of the staff if RDs, nutritionists, or dedicated medical personnel are not available; in particular, the successful implementation of nurse-led nutrition screening programs has been reported (43, 44). Mandatory screening can be implemented by a single institution or by all hospitals in the same geographic area; for example, in Lombardy, a region of Italy with a population of more than 10 million, the regional government recently mandated nutrition screening for all hospitalized patients (45).

In countries where electronic medical records are used, nutritional screening should take advantage of them; for example, a study in the U.S. evaluated the effectiveness of embedding a nutritional screening tool in the electronic health record and showed a linear increase in the completion rate of nutritional screening from 60 to 78% in 20 months (46). From the scientific point of view, the search for the best nutritional screening tool for cancer patients is likely to be futile unless different categories of patients (i.e., divided at least by cancer type and age) are considered as separate groups.

In addition, patients and caregivers should be empowered to play an active role in nutritional self-monitoring and early recognition of malnutrition-related symptoms (47). App-based interventions are increasingly used in various chronic diseases, and represent a promising approach to improve physical activity and nutrition in cancer survivors (48). Patient empowerment in malnutrition prevention could also benefit from app-based interventions, such as systems for weight monitoring or food intake measurement.

In countries where this is not already the case, nutrition education should be made mandatory in medical school and residency programs, at least in clinical areas involved in the care of patient populations with a high prevalence of malnutrition (e.g., oncology, surgery, and internal medicine). Universities should offer postgraduate nutrition courses and the creation of *ad hoc* clinical nutrition residency programs should be considered in the countries where they do not exist. Institutions should also aim to enhance the nutritional skills and responsibilities of all healthcare professionals involved in cancer care, such as nurses, through professional update courses, continuing education programs, and practical training. E-learning has been used successfully in nutrition education, even for complex scenarios such as teaching parenteral nutrition prescribing to pediatric residents at the beginning of their training, and is a promising approach to disseminating nutrition knowledge among care providers (49).

If possible, clinical nutrition specialists should be included in MTBs, at least for diseases with a high prevalence of malnutrition (e.g., pancreatic cancer, gastric cancer).

Sustainable educational interventions to improve malnutrition recognition and coding according to the DRG system should be implemented in all hospitals with DRG-based reimbursement systems, especially in the oncology departments where the prevalence of malnutrition is high. Institutions should perform adequate monitoring to ensure that malnutrition diagnoses are not lost; nutritional screening tools embedded in electronic medical records could help identify patients with a possible malnutrition diagnosis and provide a useful tool for retrospectively evaluating the accuracy of DRG coding.

Similarly, to ensure quality nutritional care for cancer patients, appropriate metrics to evaluate the effectiveness of nutritional diagnostic and therapeutic interventions should be implemented. In particular, possible evaluations include the rate of malnutrition screening among cancer inpatients and outpatients, the rate of cancer patients with significant weight loss during hospitalization, and the rate of nutritional interventions among cancer patients at nutritional risk or malnourished. In order to adequately monitor the quality of nutritional care, the recording of nutritional data (e.g., nutritional screening score, weight at admission and discharge) in medical records should become mandatory.

During the pandemic, virtual nutrition counselling was shown to be effective in improving the nutritional status of cancer patients assessed by the PG-SGA in a Turkish study (50). Other post-pandemic studies have used telemedicine for patients with mobilization difficulties, such as those receiving home parenteral nutrition, and for patient and caregiver support groups (51), although video and telephone consultations have several limitations, including the lack of anthropometric measurements and physical examination, reduced interpersonal communication, and difficulties in use by elderly patients or social groups with limited access to technology (52). Studies specifically evaluating the role of telehealth in post-pandemic nutritional oncology are lacking; however, a prospective trial on a multi-level digital health intervention for nutritional assessment and support in patients diagnosed with pancreatic cancer is ongoing (53). It is reasonable to suggest that a blended system involving both face-to-face visits (e.g., for new patient visits) and telemedicine (e.g., for follow-up visits) may be a solution for areas with a shortage of clinical nutrition specialists, due to the benefits in terms of flexibility of time, travel, and scheduling. Asynchronous online messaging systems to communicate with providers may also be an alternative. Telemedicine could also be useful in the inpatient setting, where virtual nutrition support teams could provide nutrition consultations through remote conferencing technology in multiple hospitals that lack a clinical nutrition service. This model has been evaluated for ordering parenteral nutrition for inpatients in a study conducted in the U.S., which showed a higher proportion of appropriate orders compared with the pre-intervention period (54). Unfortunately, no data are available in the literature on the cost-effectiveness and reimbursement policies of virtual nutrition visits for cancer patients.

The loss of trust in medicine is perhaps the most critical issue to be addressed due to its multifactorial origin and the broad nature of the problem, which encompasses medicine in its entirety rather than just nutritional oncology. In general, more control should be exercised over nutrition “experts” on websites and social media, perhaps requiring them to have a graduate or post-graduate education in nutrition or encouraging the reporting of “fake” experts. On the other hand, patients should be involved in educational programs to improve their media literacy and be given the tools and resources to understand the reliability of nutrition influencers. Great care should be taken by scientists who publicly promote diets or nutritional facts to maintain a high level of integrity and transparency, which are key determinants of public trust (35). In hospitals and clinics, where clinicians should strive to create the conditions to re-establish a solid and effective “therapeutic relationship” with patients. “Therapeutic relationship” is a term borrowed from the field of psychotherapy that refers to how a patient and a care provider connect, engage, and behave with each other; in the field of nutrition, a systematic review has identified several components that favorably influence relationship quality, including dietitians’ attitudes (e.g., supportive and caring) and techniques (e.g., individualizing recommendations, acknowledging client challenges) (55). Every nutrition care provider should receive psychological and communication training, which should be introduced in undergraduate courses for health professionals and updated through continuing professional development courses. On the patient side, educational initiatives (perhaps initiated by patient advocacy groups or nutrition services through webinars, brochures, group meetings, or other resources) should be implemented to provide patients and caregivers with the tools to understand and collaborate in the nutrition care process.

## 9 Conclusion

Raising awareness of the barriers to implementing nutrition care in clinical practice is fundamental, but not an easy process. Some of the issues that remain to be addressed include suboptimal nutritional screening in cancer patients, inadequate numbers of clinical nutrition staff, underreporting of malnutrition as a diagnosis in medical records, incomplete integration of clinical nutrition into multidisciplinary management, barriers to reimbursement for home nutrition support, and decreased patient trust. Multilevel interventions are warranted in the coming years, including the implementation of mandatory nutritional screening, updated reimbursement policies, educational programs and telehealth interventions.

## Data availability statement

The original contributions presented in the study are included in the article, further inquiries can be directed to the corresponding author.

## Author contributions

VP: Conceptualization, Writing – original draft. PP: Supervision, Writing – review & editing. RC: Conceptualization, Supervision, Writing – review & editing.
